# Population demographic history and population structure for Pakistani Nili-Ravi breeding bulls based on SNP genotyping to identify genomic regions associated with male effects for milk yield and body weight

**DOI:** 10.1371/journal.pone.0242500

**Published:** 2020-11-24

**Authors:** Saher Islam, Umesh K. Reddy, Purushothaman Natarajan, Venkata Lakshmi Abburi, Amna Arshad Bajwa, Muhammad Imran, Muhammad Yasir Zahoor, Muhammad Abdullah, Aamir Mehmood Bukhari, Sajid Iqbal, Kamran Ashraf, Asif Nadeem, Habibur Rehman, Imran Rashid, Wasim Shehzad

**Affiliations:** 1 Institute of Biochemistry and Biotechnology, University of Veterinary and Animal Sciences, Lahore, Pakistan; 2 Department of Biology, West Virginia State University, Institute, West Virginia, United States of America; 3 Department of Livestock Production, University of Veterinary and Animal Sciences, Pattoki, Pakistan; 4 Semen Production Unit, Qadirabad, District Sahiwal, Pakistan; 5 Livestock and Dairy Development Department, Government of the Punjab, Lahore, Pakistan; 6 Department of Parasitology, University of Veterinary and Animal Sciences, Lahore, Pakistan; 7 Department of Physiology, University of Veterinary and Animal Sciences, Lahore, Pakistan; University of Florida, UNITED STATES

## Abstract

The domestic Nili-Ravi water buffalo (*Bubalus bubalis*) is the best dairy animal contributing 68% to total milk production in Pakistan. In this study, we identified genome-wide single nucleotide polymorphisms (SNPs) to estimate various population genetic parameters such as diversity, pairwise population differentiation, linkage disequilibrium (LD) distribution and for genome-wide association study for milk yield and body weight traits in the Nili-Ravi dairy bulls that they may pass on to their daughters who are retained for milking purposes. The genotyping by sequencing approach revealed 13,039 reference genome-anchored SNPs with minor allele frequency of 0.05 among 167 buffalos. Population structure analysis revealed that the bulls were grouped into two clusters (K = 2), which indicates the presence of two different lineages in the Pakistani Nili-Ravi water buffalo population, and we showed the extent of admixture of these two lineages in our bull collection. LD analysis revealed 4169 significant SNP associations, with an average LD decay of 90 kb for these buffalo genome. Genome-wide association study involved a multi-locus mixed linear model for milk yield and body weight to identify genome-wide male effects. Our study further illustrates the utility of the genotyping by sequencing approach for identifying genomic regions to uncover additional demographic complexity and to improve the complex dairy traits of the Pakistani Nili-Ravi water buffalo population that would provide the lot of economic benefits to dairy industry.

## Introduction

The domestic Nili-Ravi water buffalo (*Bubalus bubalis*) is a well-used dairy animal in livestock in Pakistan because it contributes about 68% of the total milk production in Pakistan [1]. Besides this, it is more adaptive to environment such as heat, disease and environmental stress and a better converter of poor quality roughages into nutritious and quality food [1, 2]. There is a high variation in phenotypic attributes, which indicates that further enhancement might be possible. Milk yield of the Nili-Ravi breed is commonly associated with many influential factors; however, the breed has high milk potential as compared with other buffalo species in Asia [2]. This variability indicates the possibility of using genomic selection to improve milk traits in future breeding programs. Some bulls excel in certain economic traits but not all. All the selected bulls for this study have been made available for artificial insemination, and so these bulls were genetically tested for various traits deemed important in milk production and body weight [3].

Several genetic studies for milk yield exist and a meta-analysis suggested that a majority of female traits that contributed to milk yield in dairy cattle tend to be lowly heritable (0.02 to 0.04) when compared to the studies involving male animals with mean heritability estimates between 0.05 and 0.22 [4]. Though superior genetic effects in both males and females are fundamental to breed for high milk production, male genetics is more informative as breeding values of AI bulls can be estimated with lesser bias [5].

Advances in high-throughput sequencing technologies provide the ability to identify genome-wide diversity and bottlenecks in domestication. Superior gene pools can be enriched to reproduce buffalos efficiently with the existing genomic resources and plan future conservation strategies [2, 6]. Genetic control of any trait specifies the interaction of environmental and genetic variance [7]. Inbreeding could cause further loss of genetic diversity and could accumulate deleterious alleles in the genomes, thereby resulting in deterioration of the breed, reduced milk yield, calving performance, udder health, fertility and survivability [8, 9]. Inbreeding gives rise to continuous segments of homozygous genotypes that are linked to inbreeding depression [10]. For example, one percent increase in F_ROH_ (association between increased inbreeding based on ROH) was associated with 20 kg decrease in 205-days milk yield [11], and also with 0.4-days extended insemination interval in heifers [12]. Informed decisions based on molecular knowledge could result in selecting diverse and superior animals for sustaining milk production.

With next generation sequencing (NGS), genome-wide single nucleotide polymorphisms (SNPs) can be developed affordably and can be effectively used for genome analysis of various traits in unexplored livestock such as water buffalo of Pakistan. A normal dam typically conceives on average up to 5 or 6 times in whole life-span but once an elite bull is effectively identified, its ability to produce a large number of offspring in life-time can be maximized by using artificial insemination. The detection and development of genome-wide SNP markers are essential tools for mapping and analyses of quantitative traits for superior bull selection [13–15].

Recently, many genotyping by sequencing (GBS) studies have been conducted on dairy breeds because it is a highly multiplexed and simple approach to construct NGS libraries [16–23]. De Donato et al. [24] used the GBS approach for genotyping 47 cattle breeds from Africa and the United States, producing 1.4 million unique reads per animal, and identified 63,697 SNPs. The authors concluded that the GBS approach is flexible, novel, sufficiently high-throughput and capable of providing acceptable genetic markers for genomic selection and genome-wide association study (GWAS). Ibeagha et al. [25] used the GBS approach to identify population-specific SNPs for use in improving complex dairy traits. Akanno et al. [26] reported seven SNPs with significant dominance associations for birth, weaning and yearling weight. Surya et al. [27] annotated the genome-wide SNPs of *B*. *bubalis* and reported 436 SNPs in 38 genes affecting fertility and 483 SNPs in 66 genes affecting milk production. Gao et al. [20] used SNP genotyping and identified two novel SNPs associated with body measurement traits of cattle.

The present study aimed to identify the chromosome-based distribution of SNPs, resolve the genetic diversity, and examine linkage disequilibrium (LD) and population structure for use in GWAS of body weight and milk yield traits in Nili-Ravi buffalo bulls from Pakistan to determine the quantity of specific traits that they may pass to their daughters. In addition, we resolved chromosome-based population demography and bottlenecks.

## Materials and methods

The animal study was reviewed and approved by institutional guidelines of ethical review committee, University of Veterinary and Animal Sciences, Lahore, Pakistan.

### Population and phenotypes

In total, 167 bulls were randomly selected for this study comprising single generation. Out of 167 breeding bulls, 155 bulls were from Semen Production Unit, Qadirabad, District Sahiwal, Punjab, Pakistan and other animals were from various other private dairy farms of Punjab in Pakistan. Animals selected for the study had no history of genital infections [28] and had diverse physical parameters with reference to the length from shoulder to pin-bone, wither height, height at sacrum, width between angles of the hip, and girth and width of the face above the eyes. In addition, bulls were selected based on reproductive parameters (scrotal circumference and scrotal length). All animals were dewormed and vaccinated regularly for foot-and-mouth disease, black quarter and haemorrhagic septicaemia. Blood samples were collected by a competent veterinary surgeon after the regular quarantine of animals. Source and phenotypic details of bulls in the study are in S1 Table.

### Genotyping by sequencing

Genomic DNA for genotyping assay was isolated from blood samples of Nili-Ravi breeding bulls by using Qiagen columns (QIAamp DNA minikit, Qiagen, Hilden, Germany). The quantity of extracted DNA was measured by using the Qubit 3 Fluorometer with a double-stranded DNA HS assay kit (Invitrogen by Thermo Fischer Scientific, USA). GBS was performed as described [29, 30]. Briefly, total DNA from all samples was digested with *ApeKI* to reduce genome complexity. Digested fragments were ligated to common and barcode adapters with appropriate overhangs. Ligated products were then further pooled and amplified with compatible primers. Amplified sample pools constituted a sequencing “library”. After PCR reaction, pooled samples were gel-purified; a GBS “library” distribution of fragment sizes was checked by using BioAnalyzer (Agilent Technologies, USA). The GBS library was considered appropriate for sequencing reaction if the number of adapter dimers (approximately 128 bp in length) was minimal and the length of most of the genomic DNA fragment lengths was 170 to 350 bp. Templates were then quantified and diluted perfectly for sequencing reactions by using NextSeq 500 (Illumina, San Diego, CA).

Raw sequence reads were trimmed with use of Sickle (https://github.com/najoshi/sickle), adopting a minimum PHRED quality threshold of 20 in a sliding window. Then reads were demultiplexed and barcodes were removed. Trimmed reads were aligned to the *B*. *bubalis* reference sequences (www.ncbi.nlm.nih.gov/biosample/SAMN08640746) with Burrows-Wheeler Aligner [29]. Aligned reads were processed to identify SNPs by using SAMtools [31, 32] and barcode reads were handled and interpreted into a distinct sequence tag set, with a single TagCounts file generated per input file of FASTQ. We used TASSEL-GBS, an efficient bioinformatics pipeline considered for processing raw sequence dataset into a variant genotype file [33].

### Genetic diversity and population structure analysis

Expected nucleotide diversity (θ per bp) and Tajima’s D for various chromosomes were estimated with sliding-window analysis by using TASSEL v5.0 as described [34]. Estimation of fixation index (*F*_*ST*_) was based on Wright’s F statistic [35] with use of SNP and Variation Suite (SVS) v8.1.5. For quantitative assessment of the number of groups in the panel, we used a Bayesian clustering analysis with a model-based approach implemented in STRUCTURE v2.2 [36]. This approach involves use of multi-locus genotypic data to assign individuals to clusters or groups (K) without prior knowledge of their population affinities. The program was run with SNP markers for K values 1 to 9 (hypothetical number of subgroups), with 100,000 burn-in iterations, followed by 500,000 Markov Chain Monte Carlo iterations for accurate parameter estimates with a high-performance cluster. To verify the consistency of the results, we used 3 independent runs for each K. An admixture model with correlated allele frequencies was used. The optimal K value was determined by use of an ad-hoc statistic, ΔK [37]. The number of *K*s in each dataset was evaluated by ΔK values estimated with the software Structure Harvester, a website and program for visualizing STRUCTURE output and implementing the Evanno method. In a second approach, we used principal component analysis (PCA) with SVS v7.7.6 (Golden Helix, Inc., Bozeman, MT, www.goldenhelix.com). Female genetics was not included in the present study, which is in similar lines with several reports [3, 38–42].

### Linkage disequilibrium (LD) analysis

For GBS study, we considered only SNPs that were successfully mapped to the *B*. *bubalis* whole genome sequence (WGS) draft to avoid spurious LD and incorrect association mapping. Before analyzing LD distribution, a block of the haplotypes was called for all adjacent SNPs by using SVS v8.1.5. Adjacent and pairwise measurements of LD were calculated separately for each chromosome. For computing LD, we used an expectation-maximization (EM) algorithm, formalized by Dempster et al. [43], an iterative technique for obtaining maximum likelihood estimates of sample haplotype frequencies.

### Identification of runs of homozygosity (ROH)

ROHs were estimated using SNP and Variation Suite (SVS) that uses a sliding window to identify both ROHs and consecutive stretches that contain a minimum number of homozygous SNPs, at a minimum pre-specified distance. With this method, the software carries out basic detections of homozygous stretches identified by the sliding window, and the user only needs to define the parameter “minimum size” of segments to be identified [44].

### GWAS mapping

GWAS comprises a multiple-locus mixed linear model that utilizes a backward and forward stepwise manner to identify genetic markers in the model as fixed-effects covariates and applied in SVS v8.1.5 [45]. Manhattan plots were evaluated in Genome Browse v1.0 (Golden Helix) for significantly associated SNPs. The SNP *P*-values from GWAS underwent sequential Bonferroni adjustments [46] and false discovery rate (FDR) study [47, 48]. Gene ontology and annotation terms for the SNP-containing sequences for each trait were identified with the WGS draft of the *B*. *bubalis* genome assembly.

## Results

### Genotyping of water buffalo collection and variation identification

We genotyped 167 water buffalo samples by GBS, which generated approximately 3.71 billion reads of 75 bp long. The median number of reads for each sample was 1.5 million (range ~100K to 11.0 million). Good barcoded tags with at least 10 read counts, corresponding to ~1.75 billion reads, were obtained and used for SNP calling. The 1.75-billion GBS reads were aligned to the *B*. *bubalis* reference genome (version 1; https://www.ncbi.nlm.nih.gov/genome/791?genomeassemblyid=374666), with 98% of the unique tags mapped to the genome. From the alignments, we identified 114,760 SNPs. After retaining SNPs with ≤5 0% missing data (representing at least 617 accessions) and minor allele frequency (MAF) ≥ 0.01 (i.e., SNPs present in at least 7 accessions), we obtained 24,319 SNPs distributed across the *B*. *bubalis* reference genome, with an average of one SNP per 10.6 kb. Only eight regions >500 kb in the reference genome were not covered by SNPs, because these eight regions were centromeric or pericentromeric. The MAF distribution of these SNPs is shown in S1 Fig. Among the 24,319 SNPs with ≤ 50% missing data and MAF ≥ 0.01, only biallelic SNPs were retained in the final SNP dataset.

### Chromosome-wise SNPs

When screening for 70% call rate with 0.05 MAF, we isolated 13,039 SNPs for the 167 bulls studied; 659, 482, 507, 417, 847, 254, 317, 270, 285, 4566, 231, 230, 279, 694, 166, 236, 293, 149, 220, 145, 124, 158, 189, 89 and 1229 were mapped to the WGS draft and located on chromosomes 1, 2, 3, 4, 5, 6, 7, 8, 9, 10, 11, 12, 13, 14, 15, 16, 17, 18, 19, 20, 21, 22, 23, X and Y, respectively.

### Genetic diversity

**Fig 1:**
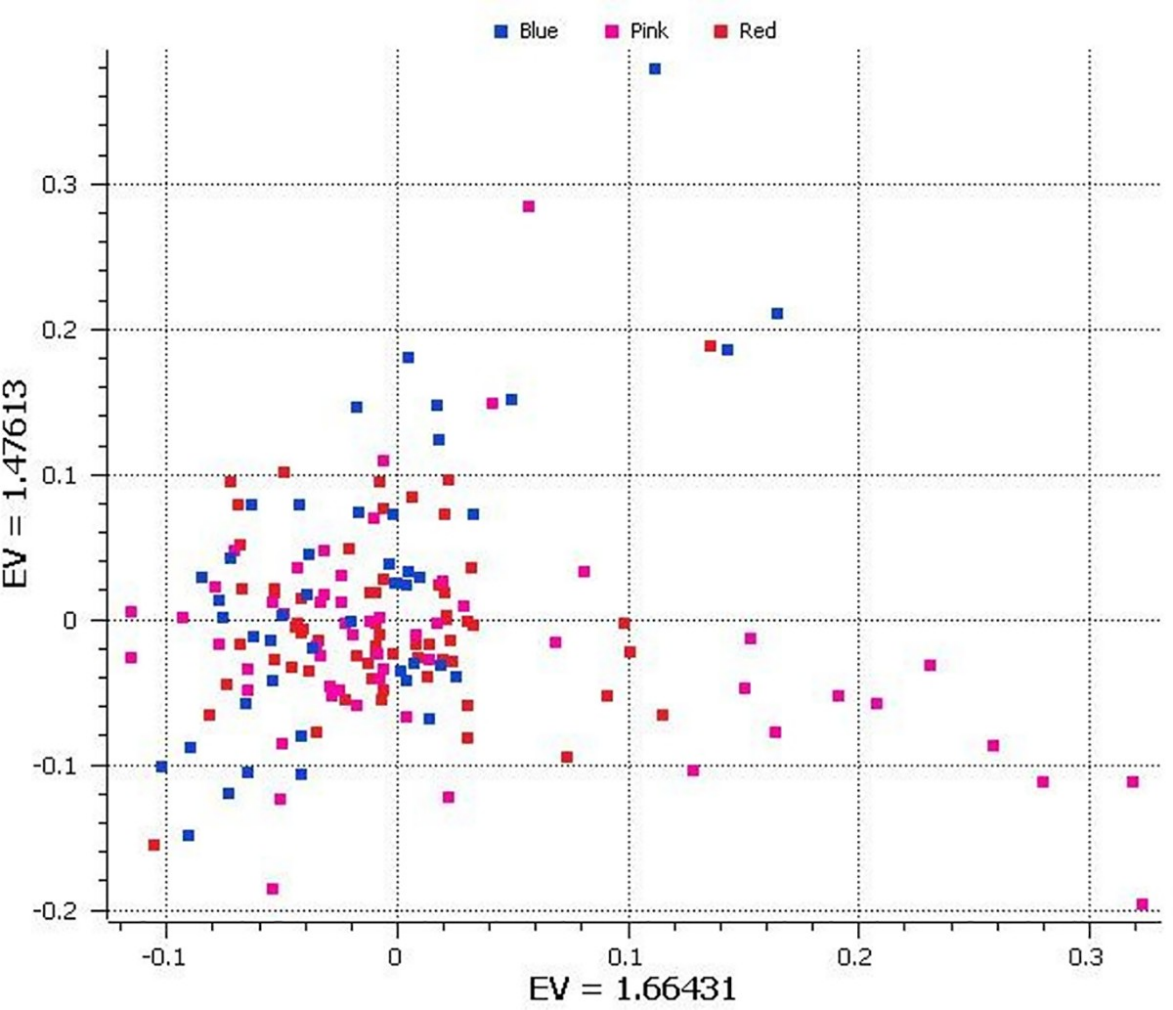
Principal component analysis (PCA) of 13,039 single nucleotide polymorphisms (SNPs) in 167 Pakistani Nili-Ravi breeding bulls. Colors are representative of high (blue), medium (pink) and low (red) milk yield bulls, majorly (93%) based on Semen Production Unit, Qadirabad, District Sahiwal, Pakistan while others are collected from different dairy farms of Punjab in Pakistan. Each point represents an individual animal and position of the points can be identified using Eigen values in S2 Table.

Using the final SNP dataset, we performed PCA, which illustrated two clusters and some outliers (Fig 1 and S2 Table). Our results are consistent with those from Lu et al. [49], who also classified water buffalos into two primary groups, with some outliers. To validate the results of PCA, we analyzed the population structure of the water buffalo with the Bayesian clustering algorithm implemented in the STUCTURE program.

**Fig 2:**
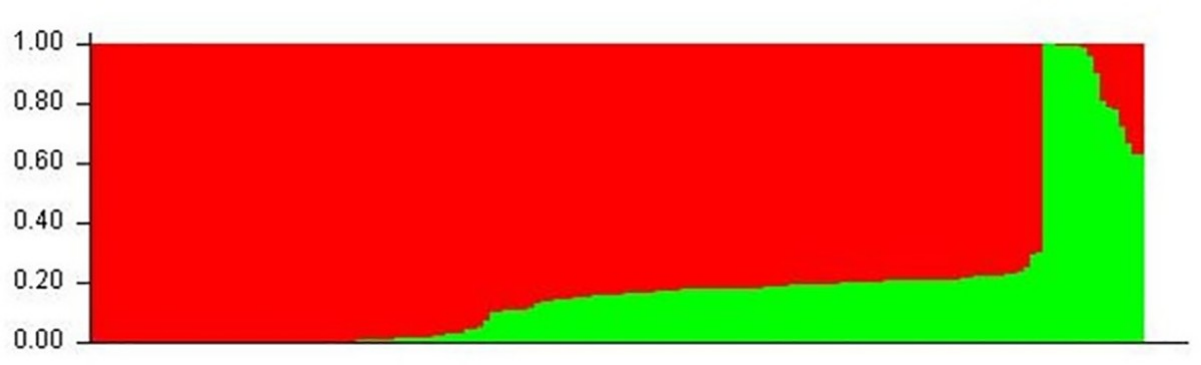
Two clusters inferred from the Pakistani Nili-Ravi population by using STRUCTURE analysis.

*ΔK* analysis showed that two populations (K = 2) represented the best number of clusters for these 167 bulls (Fig 2 and S1 Fig). As shown in Fig 2, at K = 2, bulls were grouped into two groups, which indicated the presence of two lineages in Pakistan and the extent of admixture of these two lineages (S2 Fig). The population structure at this optimal K was consistent with the PCA results; all suggested two primary clusters in the Pakistan water buffalo bull collection. These two lineages indicated that Nili-Ravi is crossbred buffalo originated from the Nili and River-type buffalos.

### Nucleotide diversity

**Fig 3:**
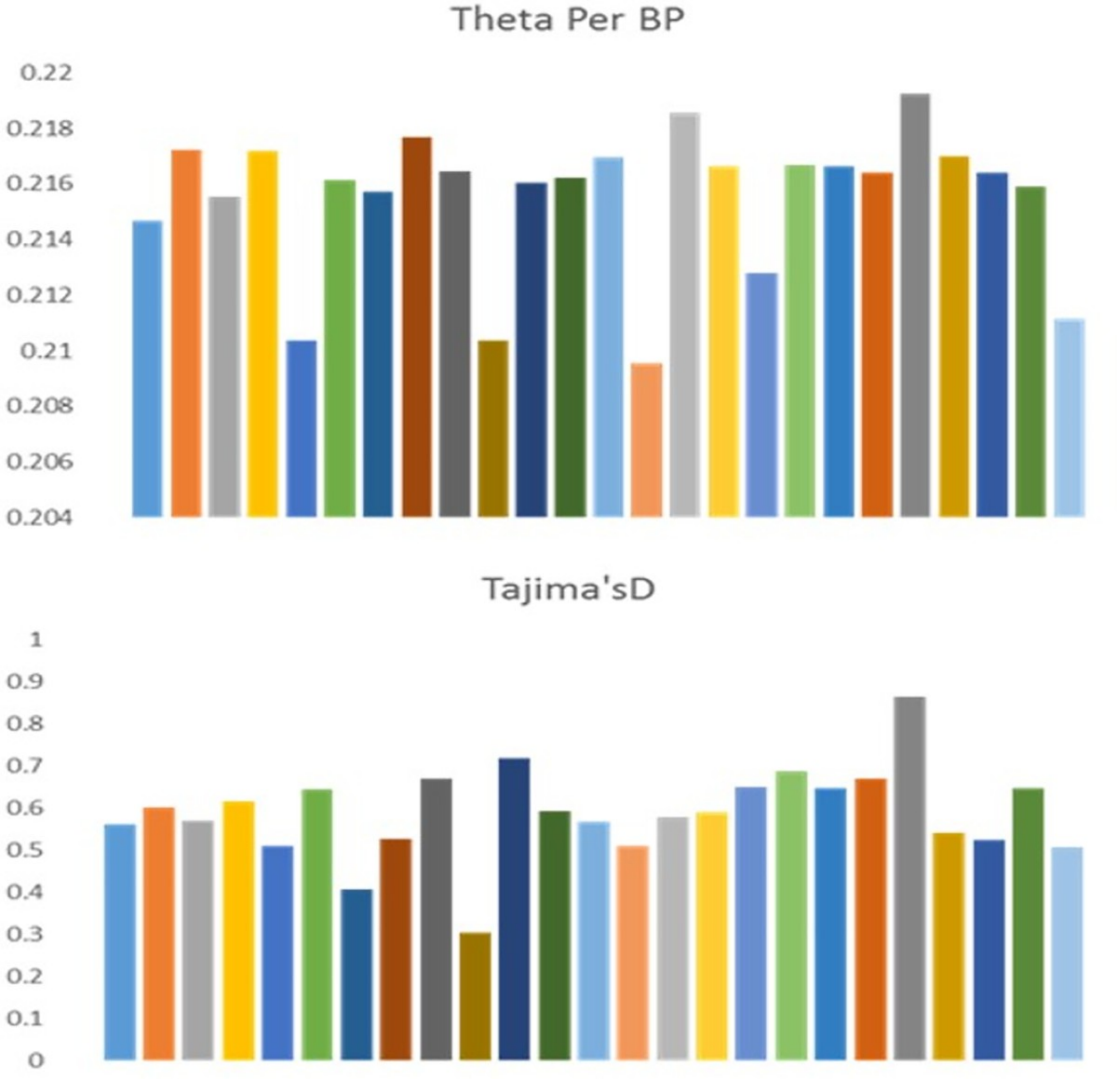
Frequency spectrum for chromosomal means for expected nucleotide diversity (θ) and Tajima’s D for Nili-Ravi breeding bulls to understand genome-wide bottleneck effects. It shows highly variable nucleotide diversity and Tajima’s D across various chromosomes.

Because of the strong population structure, we assessed patterns of expected nucleotide diversity (θ) and Tajima’s D separately for each chromosome to understand genome-wide bottleneck effects. Such information will be of immense use when inferring the evolutionary dynamics of domestication across the water buffalo genome. Water buffalo domestication is often associated with “population bottlenecks” because of the limited number of founding individuals experiencing domestication events. The frequency of segregating SNPs, as reflected by various chromosomal measures of mean θ and Tajima’s D, is presented in Fig 3. For water buffalo, chromosomes 5, 10 and 14 showed reduced nucleotide diversity as compared with the remaining chromosomes. For chromosome 10, Tajima’s D was also reduced along with mean θ, which indicates purifying selection as compared with the other chromosomes. Chromosome 21 showed increased nucleotide diversity and Tajima’s D, which indicates the accumulation of rapid mutations on this chromosome.

### LD and recombination rate analysis across the chromosomes

**Fig 4:**
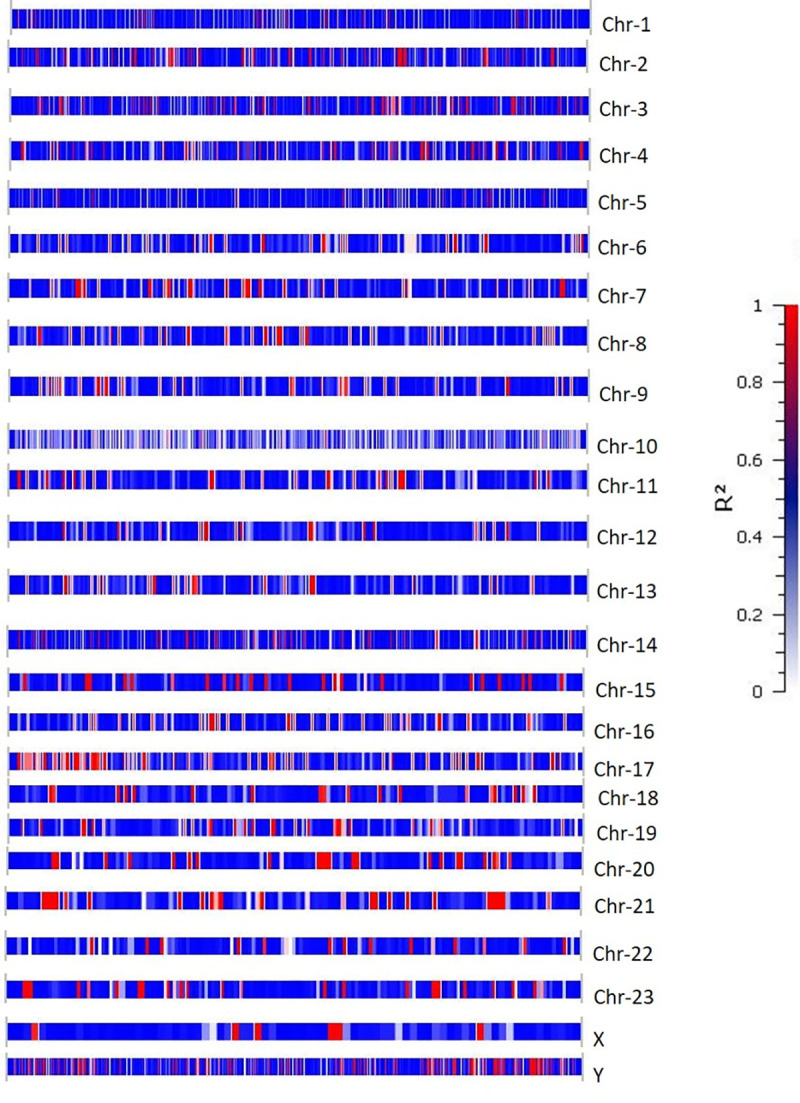
Linkage disequilibrium decay as measured by r^2^ averaged in distance intervals across the 25 Nili-Ravi bull chromosomes. Each LD bar represents a chromosome, and individual LD blocks are colored based on the scale presented.

Distribution of LD is essential to understand patterns of historical recombination and the non-random association of loci. We performed LD analysis for the whole dataset of water buffalo breeding bulls, involving all the adjacent pairs of SNPs mapped to various chromosomes (Fig 4). For computing LD, we used the EM algorithm [43] as an iterative technique for obtaining maximum likelihood estimates of sample haplotype frequencies. The number of associations mean LD block and maximum LD block sizes for various chromosomes are presented in Table 1. As expected, the largest LD block was in chromosome Y, because the Y chromosome in mammals is fixed and will not undergo recombination. In this study, we identified 4169 adjacent SNP pairs in LD, with an average LD decay at 90 kb for the Pakistan water buffalo genome.

**Table 1:** Chromosome-wise distribution of linkage disequilibrium (LD) blocks for Nili-Ravi.

Chromosome number	No. of Associations	Mean LD Block size (Kb)	Maximum LD Block size (Kb)
1	243	65.45	2068.56
2	179	100.46	3047.04
3	169	63.43	3715.18
4	156	109	1598.18
5	221	47.47	2895.55
6	100	110.17	1793.58
7	101	102.76	3821.12
8	94	109.24	2068.86
9	93	122.87	2611.62
10	1231	13.53	3259.68
11	84	103.8	3043.47
12	63	116.018	2032.92
13	98	53.43	1425.26
14	204	27.52	1445.27
15	50	117.41	2297.35
16	85	81.23	2490.50
17	132	51.64	1773.93
18	51	86.14	2075.73
19	91	56.24	2481.07
20	47	170.13	4782.65
21	52	159.45	2232.41
22	51	67.072	1182.09
23	65	150.68	3362.06
X	27	117.03	1098.19
Y	482	51.73	16734.94
Overall	4169	90.156	

### Distribution of ROH and haplotype blocks

**Fig 5:**
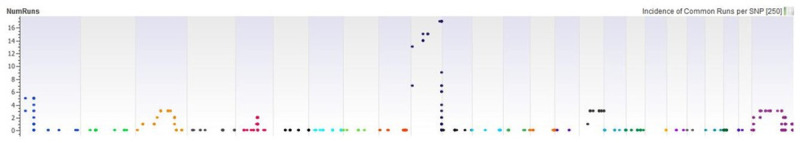
Incidence of each single nucleotide polymorphism (SNP) in a run of homozygosity (ROH) across the Nili-Ravi water buffalo chromosomes. The X-axis shows the distribution of ROH across the genome and Y-axis represents the frequency (%) of overlapping ROH shared among samples. See S3 Table for a list of ROH across all the chromosomes.

The genome-wide pattern of ROH was determined to examine local homozygosity in Pakistan water buffalo bulls (Fig 5 and S3 Table). ROH regions can be critically analyzed to identify susceptibility/deleterious loci across the genome where there has been significant evolutionary pressure. ROH regions form as a result of selective sweeps around the gene candidates that have evolutionary significance. Large regions of homozygous SNPs can be common between subpopulations without a direct common lineage, and these regions can be areas of functional significance [50]. In this study, we resolved ROH regions on chromosomes 1, 3, 5, 10, 16 and Y of varied lengths. The Y chromosome showed the highest ROH islands, which is expected, as in other mammals [51].

### Characterization of selection sweeps and domestication signature

**Fig 6:**
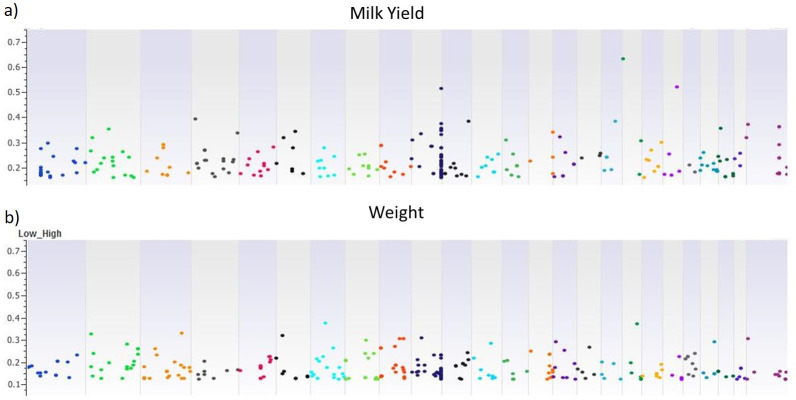
Highly significant pairwise *F*_*ST*_ (P<0.001) distribution (low vs. high) for a) milk yield and b) body weight across the Nili-Ravi buffalo chromosomes. SNPs were plotted relative to their physical positions within each autosome. Individual *F*_*ST*_ values are in S4 Table.

A highly significant pairwise *F*_*ST*_ (P<0.001) distribution with low and high milk yield and body weight for the buffalo bulls is presented in Fig 6 and S4 Table. These sliding windows allowed for identifying differential evolutionary signatures across the genome for milk yield and body weight of the bulls. Furthermore, the patterns of *F*_*ST*_ variation indicated genomic areas with the signatures of positive selection and sweeps across various chromosomes. The pairwise analysis involving low and high bull groups showed higher genetic differentiation relative to low and high milk yield rather than body weight. Selection signatures detected loci with larger effects under strong selection on chromosomes 10, 18 and 20 for milk yield with 0.4 cut-off value (Fig 6A). The sliding window of pairwise *F*_*ST*_ for low and high body-weight bulls showed higher positive selection signals on chromosomes 7 and 18 with 0.35 cut-off value (Fig 6B).

### GWAS for milk yield and body weight

**Fig 7:**
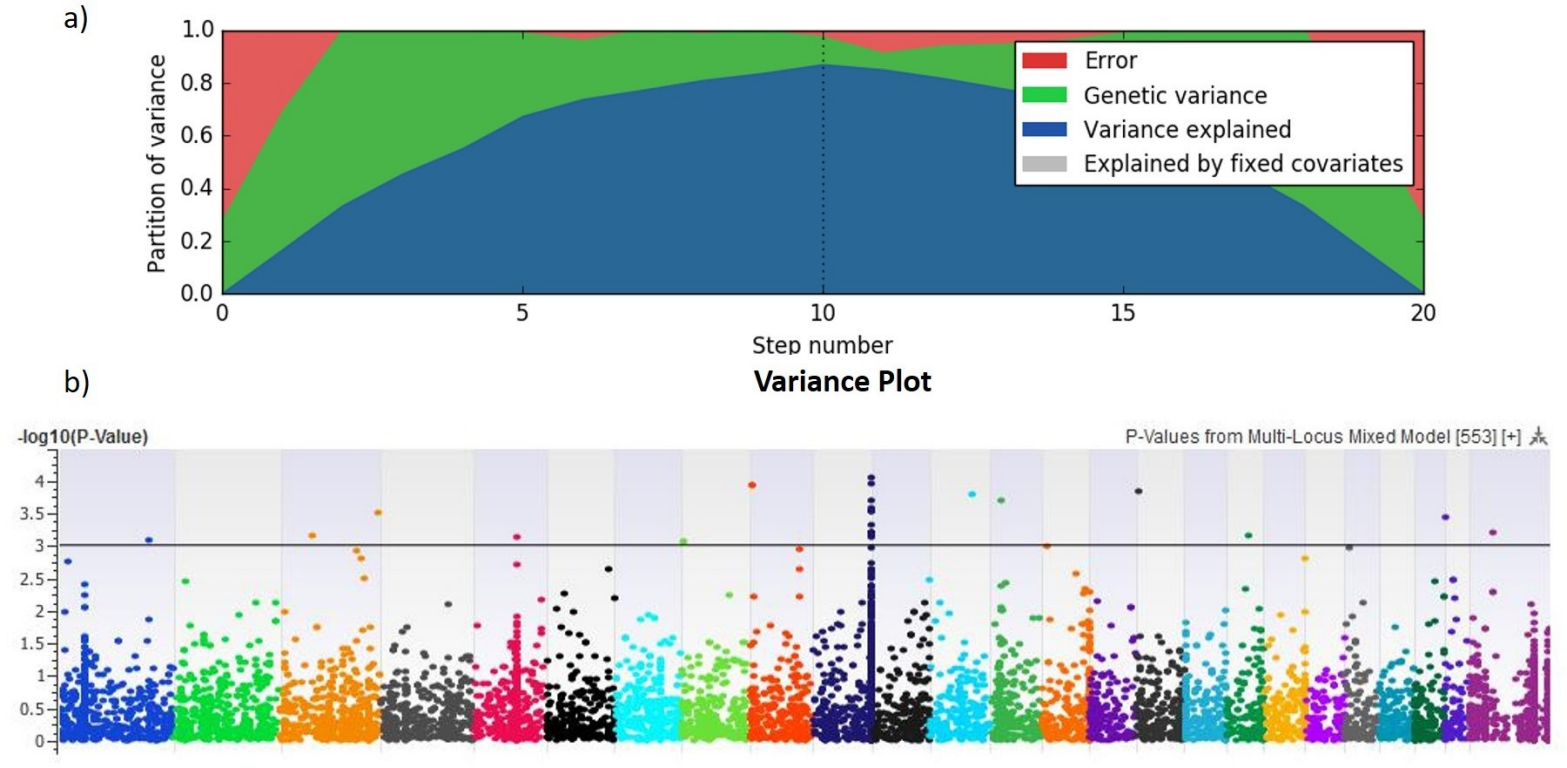
Genome-wide association results for dam's yield (liters) in Nili-Ravi buffalo. a) Variance plots partitioned error, genetic variance, variance explained in current model and variance explained by covariates. b) Manhattan plot of the chromosome coordinates on the X-axis, with the negative log-10 of the association *P*-value for each SNP on the Y-axis. High negative log-10 indicates strong association with milk yield.

**Fig 8:**
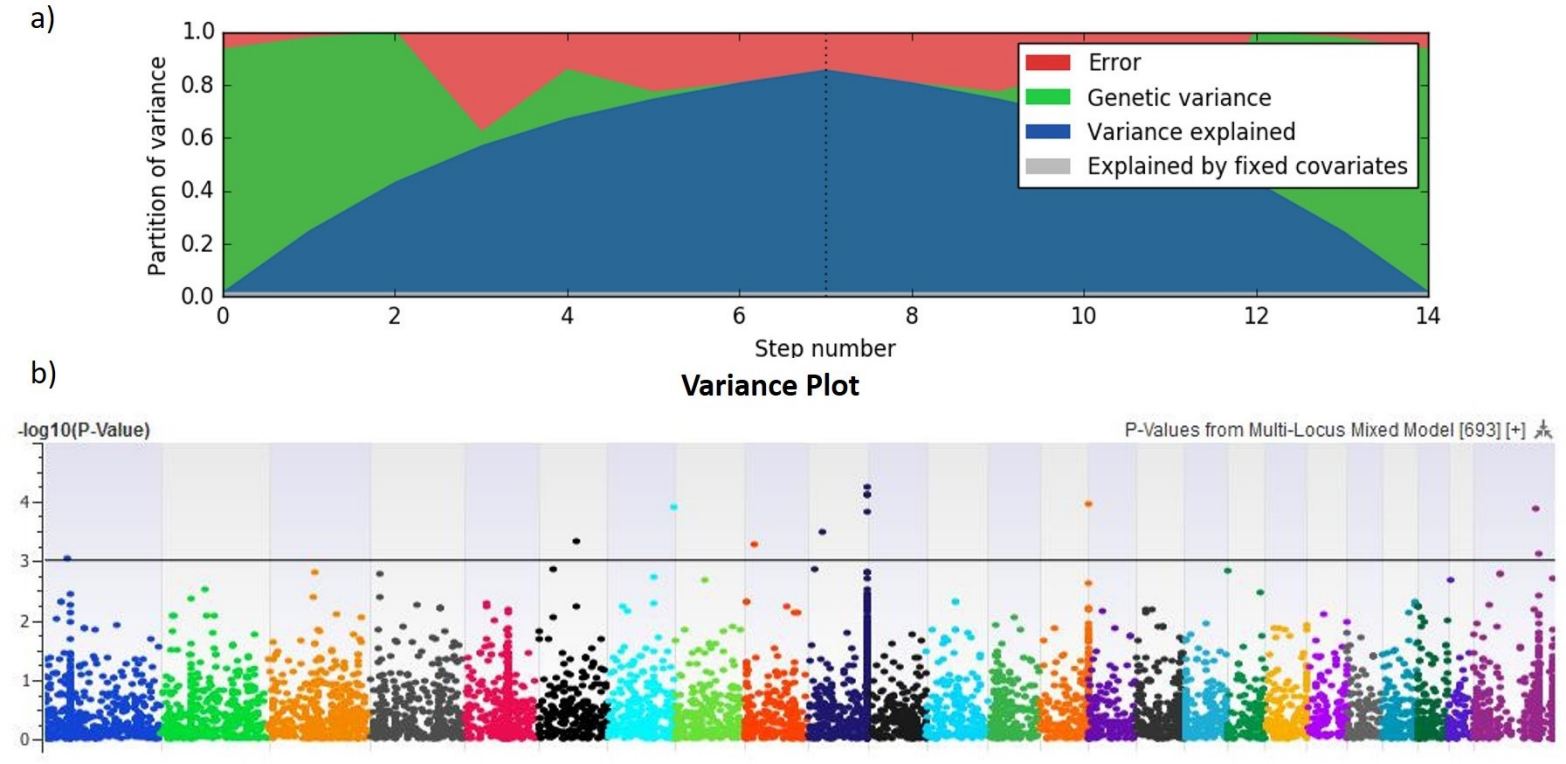
Genome-wide association results for body weight (Kg) by mixed linear model analysis. a) Variance plots partitioned error, genetic variance, variance explained in current model and variance explained by covariates. b) Manhattan plot: the black line represents the threshold at −log 10 *P*-value and plots above this line are significantly associated with body weight.

A Multi-Locus Mixed Linear model was implemented by using the EMMAX algorithm that involves stepwise regression and Byes Information Criteria (BIC) [45] to identify significantly associated SNPs for milk yield and body weight. Variance plots (Figs 7A and 8A) include partitioned error, genetic variance, variance explained in the current model and variance explained by covariates. Partition of variance explained in current study for milk yield and body weight was in the range of 70% to 80%. The–log10 *P*-values for the tested SNPs from GWAS analyses for both traits are shown as Manhattan plots (Figs 7B and 8B). All significant SNPs identified by GWAS showed cumulative phenotypic variance of 14% for milk yield and 20% for body weight. On chromosome 10, 14 SNPs were strongly associated with both milk yield and body weight. These 14 SNPs formed a tag, which is a representative in a region of the genome with high linkage disequilibrium. Such tag SNPs would reduce time and expense of mapping the genome areas associated with milk yield and body weight, because they eliminate the need to study each SNP [52]. The tag we identified on chromosome 10 explained the phenotypic variance of 14% and 22% for milk yield and body weight, respectively.

*P*-values, regression beta and minor and major allelic effects are presented for milk yield and body weight in Tables 2 and 3. Level of significance, allelic effects and phenotypic variance explained for the other milk yield-associated SNPs on chromosomes 1, 3, 5, 8, 9, 12, 13, 16, 18, X and Y are also presented. A total of 14% and 85% of the significant SNPs exhibited positive contribution for milk yield and body weight, respectively. For milk yield, significant SNPs present on chromosomes 3 and 8 participated in positive contribution. For body weight, chromosomes 1, 6, 7, 9, 10, 14 and Y positively contributed to the phenotypic variance. For body weight, each significant SNP accounted for about 16% for chromosome 1, 18% for chromosomes 6 and 9, 21% for chromosome 7, 22% for chromosome 10, and 19% for chromosome Y. Independently, all SNPs for milk yield showed a phenotypic variance of 12% for chromosomes 1, 5 and 8; 13% for chromosomes 3, 12, 18 and Y; 16% for chromosomes 9 and 16; 14% for chromosome 10 and X; and 15% for chromosome 13.

**Table 2:** Major SNPs pertaining to milk yield in Nili-Ravi.

SNP	Chr	*P*-Value	-LOG_10_ (*P*-Value)	Regression Beta	Beta Standard Error	Prop. Var. Explained	Minor Allele (Test Allele)→Major Allele	Minor Allele D Frequency
S1_158054912	1	0.00083	3.08	-1627.16	468.80	0.13	T→A	0.06
S3_172820617	3	0.000322	3.49	-1768.04	470.74	0.15	T→C	0.06
S3_55703175	3	0.000724	3.14	791.81	225.39	0.13	G→A	0.39
S5_76106561	5	0.000757	3.12	-1092.39	312.16	0.13	T→C	0.09
S8_2495291	8	0.000884	3.05	829.70	240.40	0.13	T→A	0.49
S8_2495296	8	0.000884	3.05	829.70	240.40	0.13	G→C	0.49
S8_2495349	8	0.000884	3.05	829.70	240.40	0.13	C→T	0.49
S9_4493004	9	0.000118	3.93	-1888.13	466.93	0.17	C→G	0.07
S9_4493022	9	0.000118	3.93	-1888.13	466.93	0.17	T→C	0.07
S10_104237148	10	0.000654	3.18	-1216.83	343.36	0.13	T→G	0.06
S10_104295834	10	0.000627	3.20	-1031.78	290.09	0.13	A→T	0.15
S10_104295869	10	0.000627	3.20	-1031.78	290.09	0.13	T→G	0.15
S10_104332442	10	9.22E-05	4.04	-1521.84	369.98	0.17	C→G	0.07
S10_104379763	10	0.000744	3.13	-930.90	265.64	0.13	G→A	0.16
S10_104401718	10	0.000302	3.52	-1037.95	274.97	0.15	T→A	0.15
S10_104401719	10	0.000302	3.52	-1037.95	274.97	0.15	A→T	0.15
S10_104401762	10	0.000302	3.52	-1037.95	274.97	0.15	C→G	0.15
S10_104410613	10	0.000207	3.68	-1377.10	354.59	0.16	C→G	0.07
S10_104422767	10	0.000481	3.32	-1731.56	476.12	0.14	A→T	0.08
S10_104434319	10	0.000632	3.20	-948.36	266.81	0.13	A→G	0.23
S10_104440997	10	0.000264	3.58	-1297.72	340.24	0.15	T→C	0.08
S10_104484172	10	0.000111	3.95	-1278.47	314.77	0.17	C→A	0.11
S10_104514430	10	0.000726	3.14	-1077.40	306.76	0.13	A→G	0.11
S12_75087502	12	0.000166	3.78	-1688.66	427.71	0.16	T→G	0.07
S13_18088155	13	0.00021	3.68	-1449.74	373.59	0.16	T→C	0.05
S16_3831231	16	0.00015	3.83	-1866.75	469.36	0.16	G→A	0.05
S18_39186829	18	0.000708	3.15	-1045.17	296.96	0.13	T→C	0.09
S24_1645	24	0.000372	3.43	-1418.84	382.08	0.14	C→T	0.06
S25_42968291	25	0.000649	3.19	-1235.26	348.32	0.13	G→T	0.08

**Table 3:** Association results pertaining to body weight in Nili-Ravi.

SNP	Chr	*P*-Value	-LOG_10_	Regression Beta	Beta Standard Error	Prop. Var. Explained	SNP allele	Minor Allele Frequency
			(*P*-Value)					
S1_38497677	1	0.000926	3.03	151.94	43.60	0.17	G→T	0.06
S6_67621520	6	0.000469	3.33	78.10	21.10	0.19	A→T	0.26
S7_114945813	7	0.000128	3.89	136.36	33.29	0.22	G→A	0.10
S9_17523778	9	0.000528	3.28	96.66	26.38	0.18	C→T	0.15
S10_104244729	10	8.13E-05	4.09	119.56	28.27	0.23	G→C	0.15
S10_104244743	10	8.13E-05	4.09	119.56	28.27	0.23	C→T	0.15
S10_104244754	10	8.13E-05	4.09	119.56	28.27	0.23	A→T	0.15
S10_104303781	10	5.74E-05	4.24	-146.72	33.88	0.24	T→G	0.11
S10_104392764	10	0.000151	3.82	95.22	23.53	0.21	C→A	0.35
S10_26780436	10	0.00033	3.48	83.16	21.83	0.19	T→A	0.19
S14_83340556	14	0.000112	3.95	140.95	34.09	0.22	G→T	0.13
S25_110079376	25	0.000134	3.87	141.97	34.77	0.22	A→T	0.41
S25_115449301	25	0.000756	3.12	-106.37	29.96	0.17	C→A	0.18

Some of the important gene candidates associated with milk yield were gamma-secretase subunit APH-1B pseudogene/Rho guanine nucleotide exchange factor 26 (S1_158054912), 26S proteasome non-ATPase regulatory subunit 5 (S3_172820617), Coilin (S3_55703175), uncharacterized LOC112586670/3-beta-hydroxysteroid-Delta(8), Delta(7)-isomerase pseudogene (S8_2495291, S8_2495296 and S8_2495349), kelch-like family member 29 (S12_75087502), multidrug resistance-associated protein 4-like (S13_18088155), fatty acid desaturase 2-like protein FADS2P1/olfactory receptor 5G3-like (S16_3831231), MARVEL domain-containing 3/U6 spliceosomal RNA (S18_39186829), MAPK-interacting and spindle-stabilizing protein-like (S24_1645) and EF-hand domain-containing 2 (S25_42968291). Apart from the strongly associated tag on chromosome 10, other SNPs associated with body weight were on chromosomes 1, 6, 7, 9, 14, X and Y. Levels of significance, allelic effects and phenotypic variance for the body weight-associated SNPs are also presented in Table 3. Notable candidate genes in the genomic regions associated with body weight were alpha-N-acetylgalactosaminide alpha-2,6-sialyltransferase 3 (S6_67621520), U6 spliceosomal RNA/Bardet-Biedl syndrome 7 (S7_114945813), FAM172A family with sequence similarity 172 member A (S9_17523778), glutaredoxin-related protein 5, mitochondrial (S14_83340556), spermatid nuclear transition protein 3-like (S25_110079376) and Bombesin receptor subtype 3/Protein CXorf40A-like (S25_115449301).

## Discussion

Nili-Ravi bulls are currently classified as a half herd and are an important source of milk in Pakistan [2, 53]. Because of the narrow gene pool among this herd, transferring superior alleles to progeny is important. Previous genotyping methods using low density marker systems had limitations in understanding admixture and genome-wide diversity [54]. This is the first report of the use of genome-wide SNPs generated using GBS to estimate population structure and demographic history across various chromosomes in Nili-Ravi bulls. In addition, this study is important to breeders because we identified several genomic regions associated with milk yield and body weight that could help in genetic improvement of these important economic traits. Boison et al. [55] summarized various advantages of using genomic methods for breeding buffalo bulls.

Among the 167 animals included in this study, we noted two different lineages based on admixture analysis. Similar findings were also found by Lu et al. [49] for Chinese indigenous buffalo breeds. We further noted highly variable nucleotide diversity and Tajima’s D across various chromosomes, which also agrees with previous studies of Asian buffalo breeds that used microsatellite [56–58] and mitochondrial markers [59, 60]. For example, chromosome 10 showed reduced nucleotide diversity and Tajima’s D, and chromosome 21 indicated higher nucleotide diversity and Tajima’s D.

Parameters such as diversity, Tajima’s D pattern, LD decay and ROH address various aspects of population demography and help in understanding the dynamics of genome-wide bottleneck effects for predicting genetic gain for commercial traits. LD is a random association of alleles in subpopulations and is an important parameter for LD mapping or GWAS [61–64]. One description for such varying LD would be the “Bulmer effect,” whereby eminent LD-carrying regions are mostly clustered with unique sweeps harboring crucial genes underlying domestication [65, 66]. In this study, LD analysis revealed 4169 significant SNP associations, with an average LD decay of 90 kb for the Pakistani Nili-Ravi population. We observed the maximum LD score for chromosome 10 and largest LD block for chromosome Y. Similar results observed the LD decline at approximately 50 kb, with an average *r*^2^ ≥ 0.2 in Chinese crossbred buffalo groups [Jianghan × Nili-Ravi × Mediterranean and Nili-Ravi × Murrah × local (Fuzhong or Xilin)] [67] and an *r*^2^ value from 0.412 to 0.139 in Indian Nili-Ravi buffalo [68], which is much lower than that found in our study. However, comparing our findings directly with previous reports is difficult because of differences in the scale of sampling, given that the level of LD decreases with increasing sample size [69]. Recently, the pattern of LD decay was reported in various cattle and buffalo breeds across the world, including an Iranian water buffalo population at an average of 57 kb decay [70], Chinese buffalo breeds at 50 kb for the river group and 15 kb for the swamp group [49], Tunisian cattle at 73.4 kb [71], Korean cattle breeds from 26 to 32 kb [72] and Colombian Creole cattle breeds from 70 to 100 kb [73].

We performed GWAS using genome-wide SNPs for several novel candidate genes related to milk yield and body weight traits that are significant breeding goals for dairy industry. Gamma-secretase subunit APH-1B pseudogene/Rho guanine nucleotide exchange factor 26 gene (Gene ID: S1_158054912) present on chromosome 1 encodes a large protein that functions as a GDP-to-GTP exchange factor and is reported to be a significant candidate gene for the maternal effect on weaning weight and milk yield traits in cattle [74–76]. In this study, chromosome 3 harbored at least two genes that affect milk yield. The first is 26S proteasome non-ATPase regulatory subunit 5 (Gene ID: S3_172820617). The second is Coilin (Gene ID: S3_55703175) and is one of the main molecular components of Cajal bodies. Both of these gene members are involved in protein-mediated degradation found in mammary glands of cows, and this mechanism is involved in milk production of dairy cows [77, 78]. The formation of various cellular bodies and small nuclear ribonucleoproteins depends on specific structural proteins, such as Coilin in Cajal bodies [79, 80]. A subset of nuclear bodies uses specific long noncoding RNA (lncRNA) as their scaffolding molecule and is required for mammary gland development and lactation [81, 82].

Uncharacterized LOC112586670/3-beta-hydroxysteroid-Delta(8) is one of the major enzymes that participates early in lactation in dairy cattle. An lncRNA (LOC112586670) fragment is a candidate gene associated with buffalo milk production [49, 74, 83–86]. From functional analysis of liver microarray data from mid-lactating Holstein cows, delta(7)-isomerase pseudogene (Gene ID: S8_2495291, S8_2495296, S8_2495349) is involved in the cholesterol biosynthesis pathway [87, 88]. This finding suggests that the candidate gene may contribute to milk metabolic pathways. Many studies have reported the kelch-like family member 29 (Gene ID: S12_75087502) involved in growth [89] and milk production in dairy cattle and buffalos [78, 90, 91]. Recently, it was also identified as the closest gene mapped by novel QTL-lead SNP for milking speed in French Holstein cattle, and T was an effect allele with a −0.08 allelic substitution effect of the SNP [92]. Multidrug resistance-associated protein 4-like (Gene ID: S13_18088155) is a member of C subfamily of ATP-binding cassette (ABC) transporters. ABCC4 acts in basic metabolic pathways and is highly polymorphic, with potential effects in a variety of phenotypes, and has also been detected in milk during early lactation [74, 91, 93–95]. The genetic effects of ABCG2 polymorphism have been observed for milk yield traits in Chinese Holstein cattle [96, 97]. Alim et al. [98] reported that the A allele was the more frequent in Chinese Holstein cattle (0.53), followed by the C allele (0.47). The authors identified that the CC genotype was associated with increased milk yield and protein percentage during the first and second lactation, whereas the C-A genotype decreased protein yield, so the C allele is associated with superior milk performance [97, 98], which is in partial agreement with our findings. Multidrug resistance-associated protein 4-like may be a novel candidate gene associated with milk yield.

In the bovine, fatty acid desaturase 2-like protein FADS2P1/olfactory receptor 5G3-like (Gene ID: S16_3831231) is a potential genetic marker for fatty acid composition in cattle milk [99–101]. In Jersey cattle, the major allele was A, and significant associations were recorded for the milk lauric, behenic, lignoceric, oleic, eicosatrienoic, and docosadienoic fatty acids. In Polish Holstein-Friesian cows, the G allele was prevalent, and significant associations were observed for erucic and docosahexaenoic acids [99]. MARVEL domain-containing 3/U6 spliceosomal RNA (Gene ID: S18_39186829) could be a novel marker related to milk trait because on relative expression analysis, its closest members (RNU6B, RNU5A and RNU1A) were used to assess the suitability of a combination of small nuclear RNAs as a reference RNA in milk somatic cells of lactating yaks [91, 102, 103]. MAPK-interacting and spindle-stabilizing protein-like (Gene ID: S24_1645) is involved in regulating milk synthesis [91, 104–106]. EF-hand domain-containing 2 (Gene ID: S25_42968291) is one of the most common calcium-binding structural motifs, and a well-defined helix-loop-helix structural domain, present in many calcium-binding proteins, has been identified in milk [107–110]. Our results support these important insights into the synthesis of milk proteins and potential targets for the future improvement of milk quality.

We identified alpha-N-acetylgalactosaminide alpha-2,6-sialyltransferase 3 (Gene ID: S6_67621520) as a novel marker for body weight, and it also contributes to protein glycosylation for milk synthesis [111–113]. U6 spliceosomal RNA/Bardet-Biedl syndrome 7 (Gene ID: S7_114945813) is associated with a rare hereditary disorder that has several phenotypic features including retinopathy, obesity, polydactyly and hypogenitalism. Among them, obesity is the major clinical finding and the incidence is reported to be 72% to 86% in the Bardet-Biedl syndrome population [114, 115]. From previous findings, Bardet-Biedl syndrome 7 gene could be considered a good marker for body weight. FAM172A family with sequence similarity 172 member A (Gene ID: S9_17523778) plays an important role in cell proliferation and facilitates S-phase entry. Thus, this gene is important as a cell growth regulator [116, 117]. FAM172A is also reported as a candidate gene for CHARGE syndrome (heart defects, atresia of choanae, retardation of growth and genital abnormalities) [118]. Glutaredoxin-related protein 5, mitochondrial (Gene ID: S14_83340556) is required for normal cell growth [119, 120]. Loss of glutaredoxin 3 impedes mammary lobuloalveolar development during pregnancy and lactation [121]. Our study adds further support to the importance of this gene in relation to the body weight trait. Spermatid nuclear transition protein 3-like (Gene ID: S25_110079376) was found a new marker for body mass. Large testes in relation to body mass (relative testes mass) is a strong predictor of high sperm competition levels in many taxa [122]. This gene family member is essential in chromatin condensation during spermiogenesis and hence, the family members are candidate genes for identifying sperm motility markers [123] and for semen quality traits [124]. Bombesin receptor subtype 3/Protein CXorf40A-like (Gene ID: S25_115449301) has been studied in animals as an important regulator of body weight, energy expenditure and glucose homeostasis [125–127].

Several female fertility traits in dairy cattle showed reduced genetic correlations among milk production traits because of low or moderate heritabilities [4, 128]. The male lines received the greatest attention in selection because of accurate prediction of sire's heritability [129]. Estimated breeding values of AI bulls are considered to be more reliable for various predictions [130]. For this reason, many GWAS studies in dairy cattle has been carried out based on bulls’ performance for use in genomic selection programs. The use of female data in GS programs has raised some debatable issues such as readjustment of phenotypes for comparison with the bulls [131]. A study that identified 105 genome-wide significant SNPs for milk production traits was through a paternal transmission disequilibrium test that explored associations from the sire families [132]. In similar lines to the current study, Nayeri and Stothard [86] utilized 3,729 North American Holstein bulls for GWAS and a genome scan with 4280 Nordic Red Cattle bulls was used to identify loci affecting milk yields Touru et al. [133].

We also performed pairwise population *F*_*ST*_ (P<0.001) differentiation for low and high bulls for milk yield and body weight to understand pattern of selection [134]. All significant SNPs present on chromosomes 10 and 18, including MARVEL domain-containing 3/U6 spliceosomal RNA (S18_39186829), had high *F*_*ST*_ values indicating positive selection at these regions. Flori et al. [135] indicated reduced *F*_*ST*_ values for GHR gene in milk yield of cattle and also identified 13 highly significant genomic regions with varied pairwise *F*_*ST*_ subjected to recent and/or strong positive selection. For body weight in our study, chromosomes 7 and 18 had high positive selection signals.

Defining ROH in a complex pedigree can be a way to examine homozygosity levels across the genome that has resulted by inbreeding and selection [136]. In this study, we observed ROH regions across the buffalo genome on chromosomes 1, 3, 5, 10, 16 and Y, with varied lengths. Chromosome 10 showed the longest ROH indicating functional significance of several important milk yield associated loci. Similar results were reported in several cattle breeds: shorter ROH results from the crosses with distant haplotype relatedness, and recombination has had more time to trim down ROH that is the target of selection sweeps, whereas greater ROH length is mostly caused by recent inbreeding [137, 138]. We identified two significant SNPs (S5_76106561; S10_104237148) in ROH that were negatively associated with the milk yield trait, so those could be deleterious alleles and fixed in the population. Three other body weight associated SNPs (S10_104303781; S10_104392764; S25_110079376) are in the ROH region, S10_104303781 was negatively associated with the weight. Longer ROH had stronger linear relationship with the number of individuals that carry deleterious homozygous mutations reducing the biological fitness [51]. For example, a large number of genomic regions that contain long ROH manifested unfavorable associations because of deleterious mutations with milk yield in Holstein cattle [139].

## Conclusion

Our study demonstrates the utility of the GBS approach for genetic analysis of complex dairy traits and for understanding the population structure of Pakistani Nili-Ravi buffalos. Our SNP analysis revealed that Nili-Ravi bulls are admixed with two lineages. Our GWAS for milk yield and body weight identified SNPs located in candidate genes previously identified in other dairy breeds. Results of this study suggested that male effects contribute strong genetic effects for milk yield. In future, favorable allele combinations from this study could be used to generate Nili-Ravi breeding bulls with high milk yield and body weight by selecting the bull with higher genetic merit is tantamount that will produce a daughter, who in-turn produces the greatest net revenue for dairy industry in terms of improved or superior commercial traits. Performance of their daughters will later be used to update these measures. Since these dairy bull data were publicly available, individual bull efficiency results can be disseminated to potential semen buyers.

## Supporting information

S1 Fig Clustering assignments of Pakistani Nili-Ravi bulls based on ADMIXTURE analysis for inferred K = 2. Each individual is represented by a single vertical line divided into K colored segments, where K is the number of ancestral populations.
(TIF)

S2 Fig Delta K analysis by using STRUCTURE Harvester to identify significant clusters (K) for Pakistani Nili-Ravi buffalo bulls. (TIF)

S1 Table Phenotypic data of Nili-Ravi bulls for milk yield and body weight traits. (XLSX)

S2 Table Delta K analysis by using STRUCTURE Harvester to identify significant clusters (K) for Pakistani Nili-Ravi buffalo bulls. (XLSX)

S3 Table Distribution of runs of homozygosity (ROH) across Nili-Ravi buffalo chromosomes. (XLSX)

S4 Table Pairwise Fixation index (*F*_*ST*_) values for individual SNPs across chromosomes of Nili-Ravi genome. (XLSX)
