# Supplementary figures and images for: Population demographic history and population structure for Pakistani Nili-Ravi breeding bulls based on SNP genotyping to identify genomic regions associated with male effects for milk yield and body weight
Source: PLoS One. 2020 Nov 24;15(11):e0242500. doi: 10.1371/journal.pone.0242500 (PMC7685427; doi:10.1371/journal.pone.0242500)

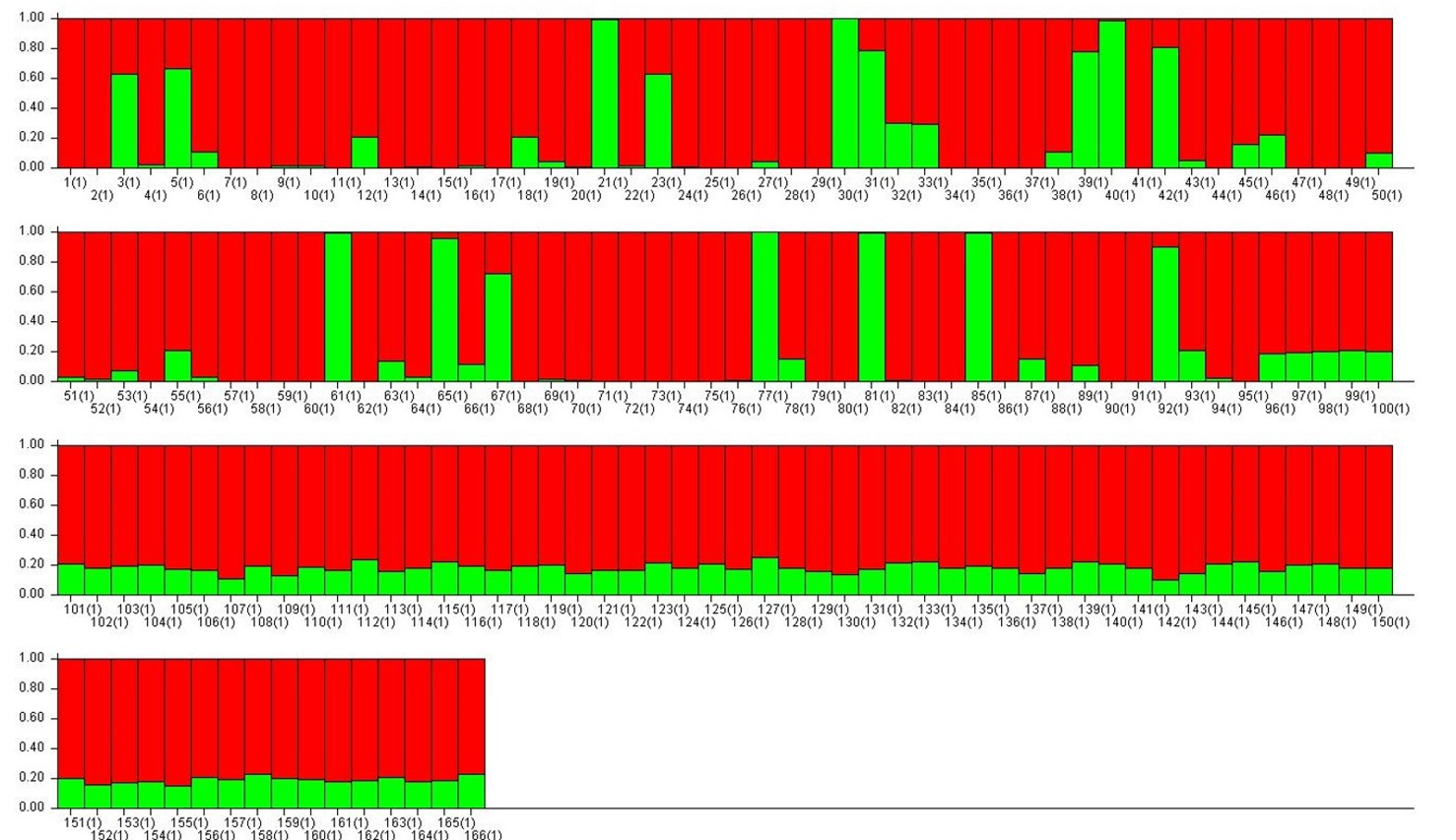

Supplement: S1 Fig — Each individual is represented by a single vertical line divided into K colored segments, where K is the number of ancestral populations. (TIF) [file pone.0242500.s001.tif]

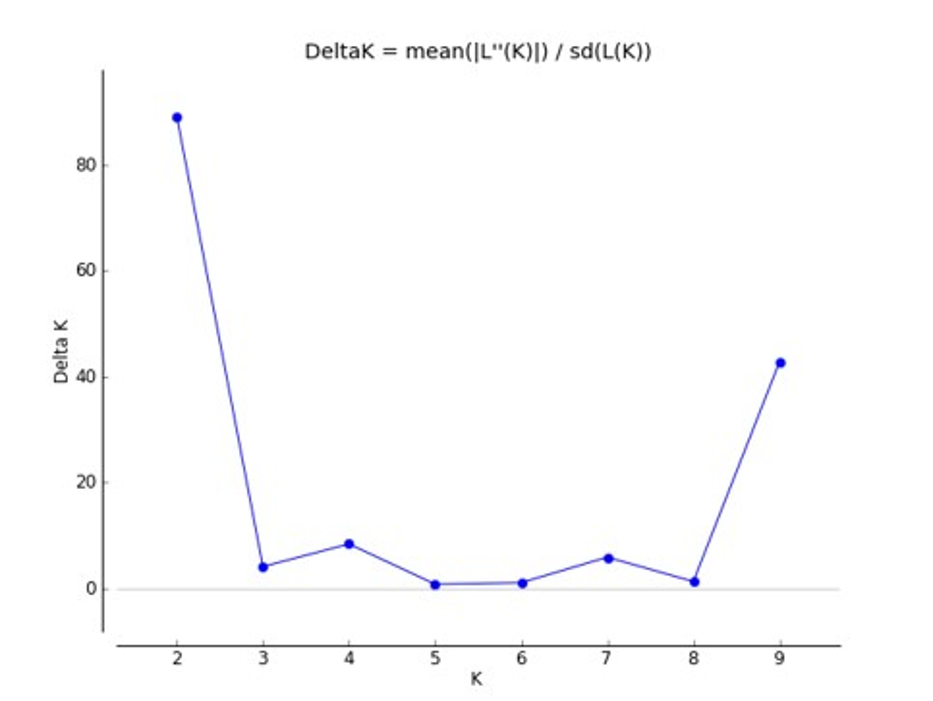

Supplement: S2 Fig — (TIF) [file pone.0242500.s002.tif]
